# Stress In An Oncologist’s Life: Present But Not Insurmountable (Students Should Not Be Discouraged Choosing Oncology as Their Future Specialisation)

**DOI:** 10.1007/s13187-015-0928-7

**Published:** 2015-10-17

**Authors:** Jonatan Nowakowski, Grazyna Borowiec, Izabela Zwierz, Wojciech Jagodzinski, Radoslaw Tarkowski

**Affiliations:** 1 Wrocław Medical University, Wybrzeże L. Pasteura 1, 50-367 Wroclaw, Poland; 2 Analiza Badania Rozwoj, Osiedle Stefana Batorego 11 lok 74, 60-687 Poznan, Poland; 3 Department of Oncology, Division of Surgical Oncology, Wroclaw Medical University, pl. Hirszfelda 12, 53-413 Wrocław, Poland

**Keywords:** Oncologists, Stress, Burnout, Coping strategies

## Abstract

Burnout is a serious problem facing the majority of oncologists. Many medical students may regard oncology as depressive part of medicine. This false picture may discourage them from choosing oncology as their future specialization. To learn problems experienced by oncologists and to answer the students’ question: is it dangerous to choose oncology? We conducted an anonymous survey among 69 oncologists. Young doctors (up to 5 years of service) accounted for 31 %, specialists 69 %, with a median length of practice of 14 years. The most frequently reported symptoms included irritability (84 %) and tension (74 %). Forty-five percent reported headaches, 25 % sleep disorders, 51 % negative impact on their personal lives. Excessive bureaucratization, overwork, and haste, with the disparity between undertaken effort and compensation were the most common sources of stress. Stress reduction methods were as follows: their relationship with family and/or friends (69 %), reading books/watching movies (66 %), emotional distance from their problems (63 %), and contact with nature (62 %). Ninety-six percent of physicians were satisfied with their choice of pursuing work with cancer patients. However, as many as 49 % of oncologists experienced moments of doubt regarding their sense of vocation. Students and young doctors considering pursuing an oncological speciality should not be discouraged by the likely degree of sacrifice or burden, but rather aim to develop effective ways to reduce stress, along with remembering one’s own health needs. This could be valuable part of both pregradual and postgradual medical education, worth to become part of medical curricula.

## Introduction

Working with oncological patients requires not only a vast interdisciplinary knowledge of modern medicine and the ability to apply it in practice but also a psychological predisposition to accompany the suffering patient and the ability to counter the harmful stress to which the medical professional is exposed. Recent scientific reports are alarming: a study published in 2014 at the ESMO Congress reports the presence of burnout symptoms among as many as 71 % of young oncologists in Europe (Central Europe 81 %) [1], while the percentage of burnout among US oncologists reaches 45 % [2], and 34 % of oncologists working in the USA report the urge to change jobs within the next two years [3]. The practical aim of our pilot study was to present the problems experienced by oncologists and to arouse interest in oncological specialities among medical students; given that, currently, these specialities are urgently needed. We decided to perform this study to find the answer on questions: *why* do we become oncologists and *who* are we becoming oncologists. Our study listed reasons and motives for choosing specialities among medical students and further—physicians, the presence of job satisfaction, the conversion of personal goals and values over the years, studied the level of stress, and analyzed stressors and effective stress reduction techniques.

## Methods

### Participants

The survey was conducted among oncologists working in three hospitals in Wroclaw, Poland. It included 69 participants: 36 radiation oncologists (R), 10 surgical oncologists (SO), and 23 oncological and palliative care physicians (S + P). Young doctors (up to 5 years of service) accounted for 31 % (*n* = 21), specialists 69 % (*n* = 47) with a median length of practice of 14 years. One person did not report neither the age nor the experience. Women accounted for 64 %, men 36 %.

### Measures

**Fig. 1:**
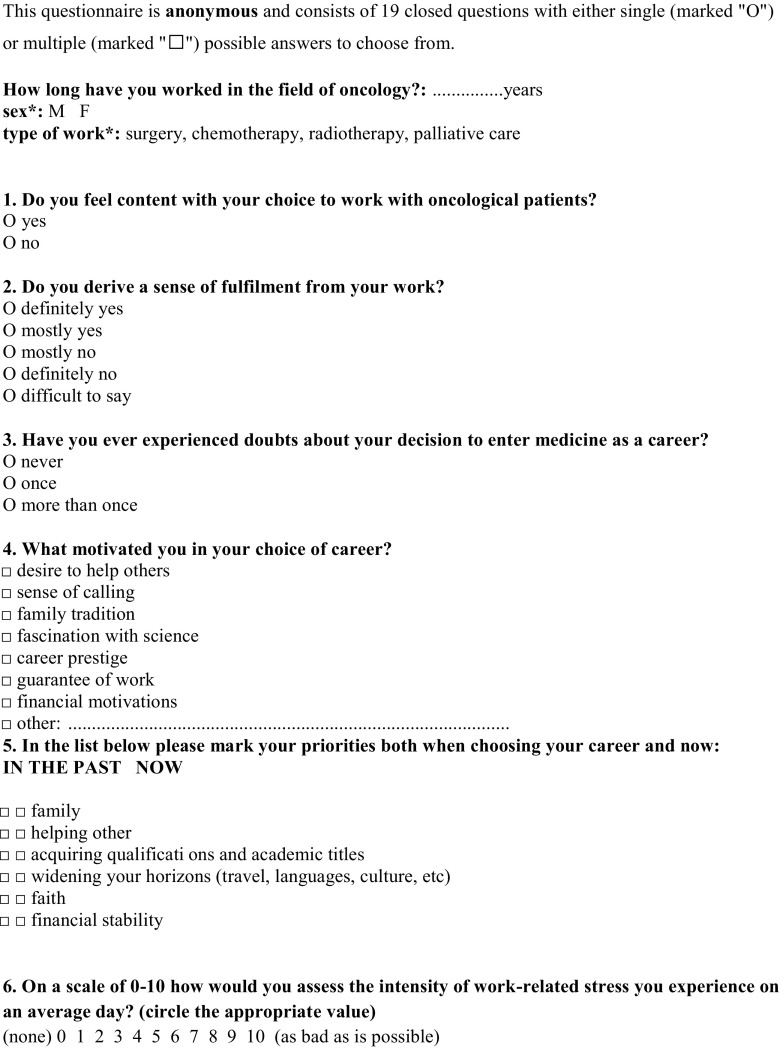

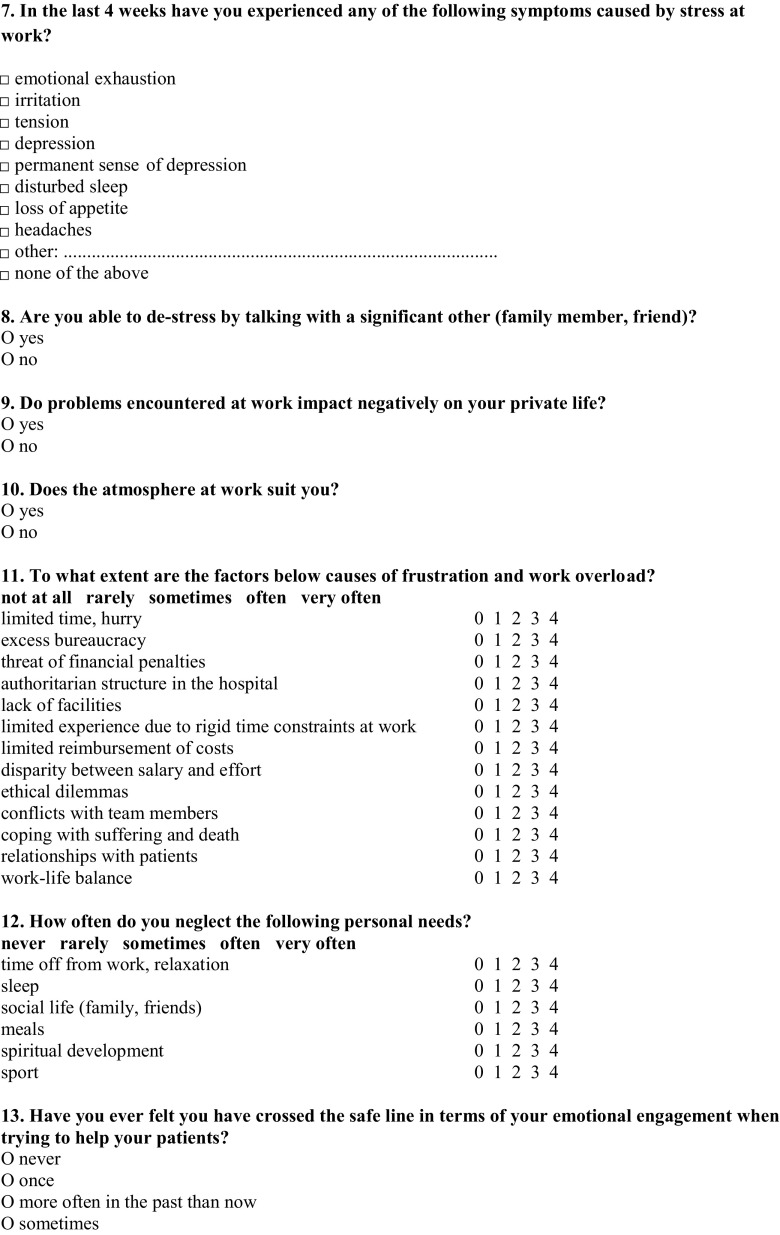

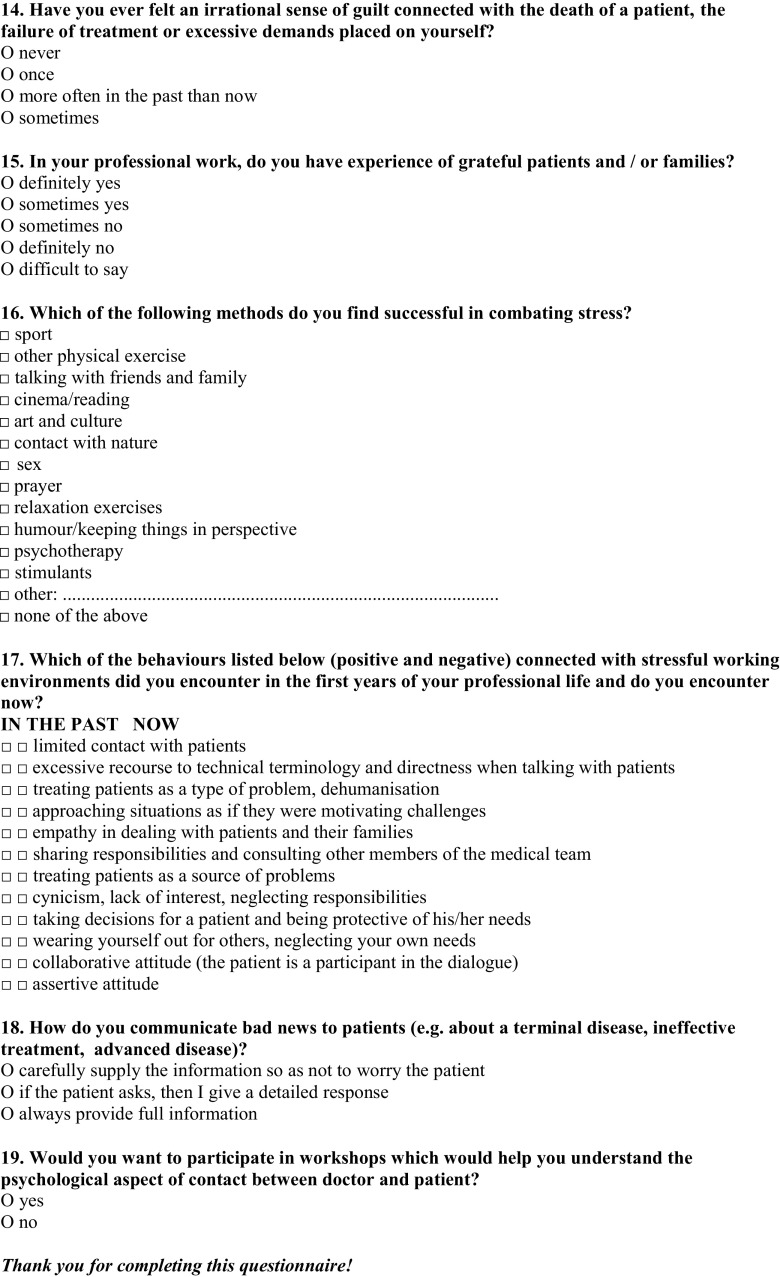
Questionnaire for oncologists

The self-constructed, confidential, and anonymous survey consisted of two parts: demographic data (seniority, specialization, gender) and 18 questions regarding the subject of the study. The survey was not tested. There were 14 closed questions with one correct answer, 5 with multiple answers: two of them closed and three with further possibility of writing one’s own interpretation of an answer (Fig. 1).

**Fig. 2:**
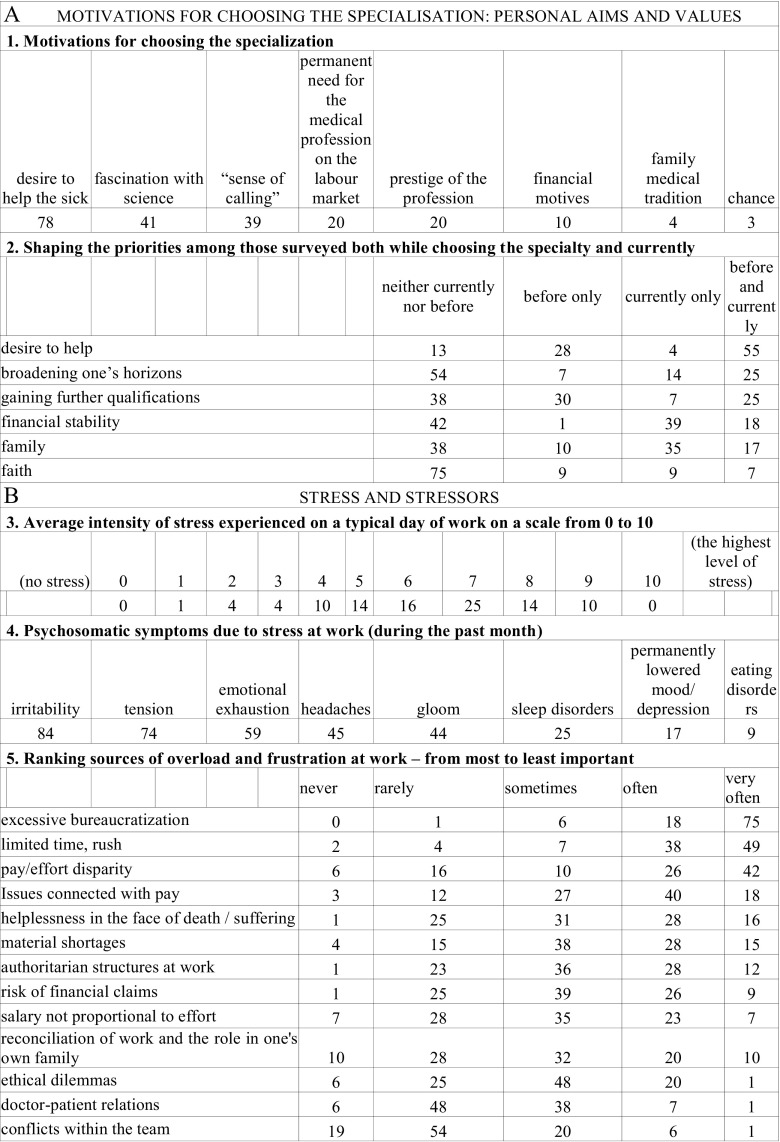

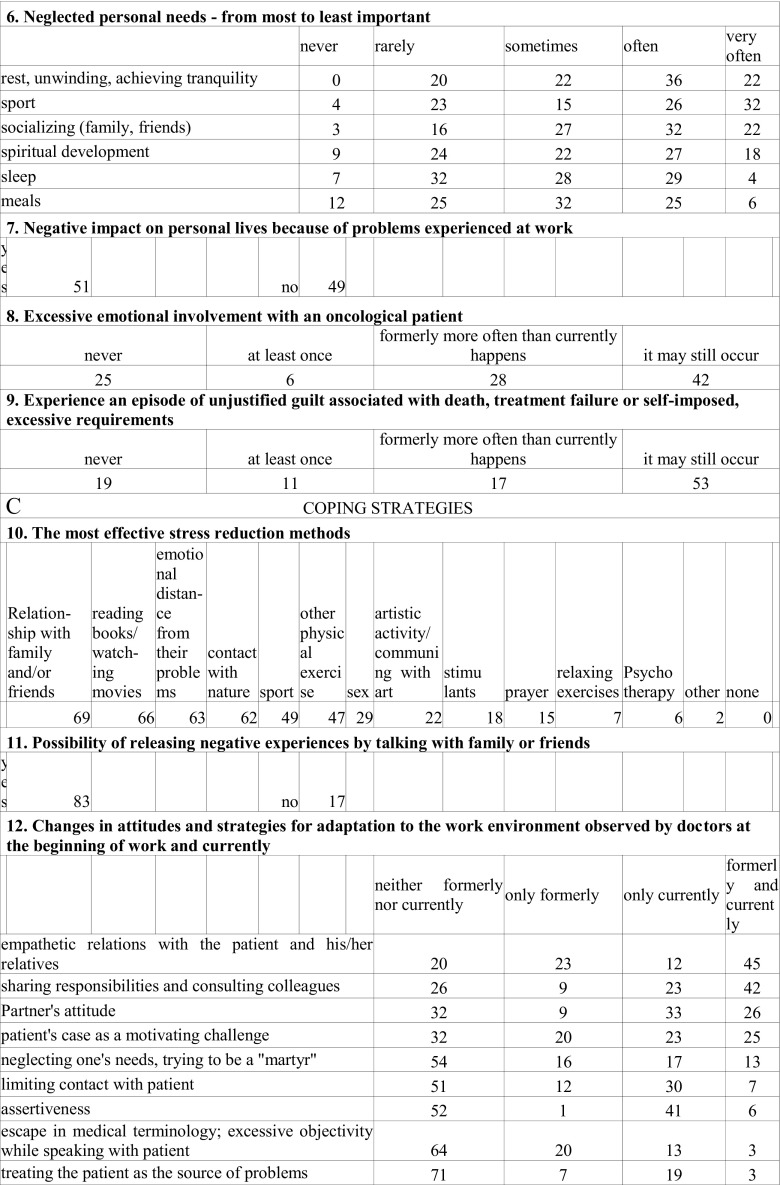

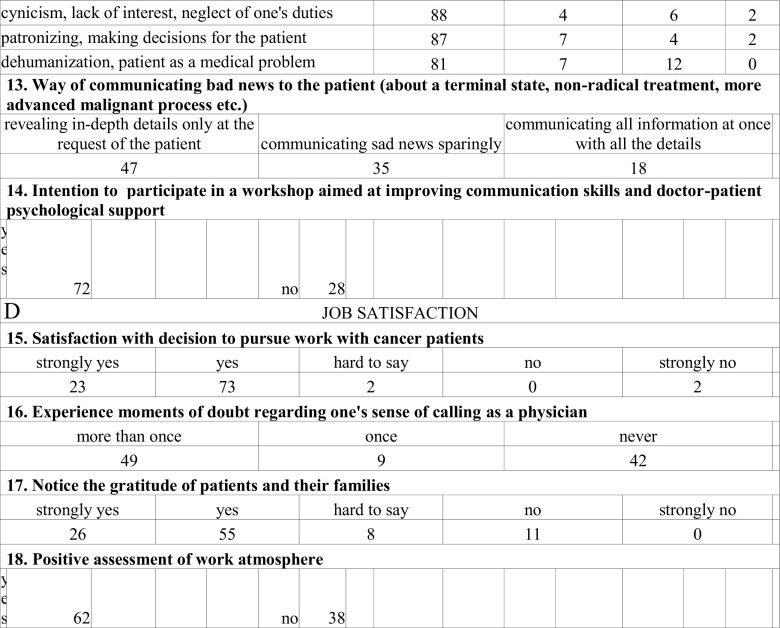
Survey questions, along with a statistical analysis (%)

To prevent the identification of the place of employment, no geographical data were collected. Survey questions, along with a statistical analysis are presented in Fig. 2. The response rate was 75.5 %, including responses obtained from 71 oncologists. Two forms were further rejected due to incompleteness.

### Statistical Analysis

Due to the limited reach of the survey and small study group with a disproportionate distribution, the study focused primarily on descriptive statistics. To test hypotheses related to the level of stress shown on an 11-point and one-quotient scale, the parametric *t* test was used on a plan for two independent groups (*k* = 2) and one-way analysis of variance ANOVA for *k* > 2 independent groups. Each of these tests was preceded by a confirmation of a normal distribution (KS test), and for ANOVA by a Levene’s test of homogeneity of variance. Statistically significant results for ANOVA (*p* < 0.05) resulted in further analysis utilizing post-hoc tests (Tukey’s test), highlighting the fact that there are significant differences between specific groups.

For the dependent variables measured at the ordinal level (frequency and Likert scale), nonparametric tests counterparts were used; i.e. in the case of comparing two independent groups, Mann-Whitney *U* test (test of difference in ranks), and in the case of *k* > 2 H Kruskall-Wallis test.

Calculations were performed using the IBM PASW Statistics 21 package.

## Results

### Motivations and Values (Fig. 2A)

The strongest motive for choosing the specialization was the desire to help the sick (78 %), followed by a fascination with science (41 %), and a “sense of calling” (39 %). Thirty percent took into account the continued need for the medical profession on the labor market. The prestige of the profession, financial motives, or one’s family medical tradition proved to be less important. The desire to help weakened along with the length of seniority, just as the desire to acquire academic and professional qualifications did. Over the years of practice, there was a noticeable upward trend in the pursuit of financial stability and the importance of having a family.

### Stress (Fig. 2B)

The average intensity of stress experienced on a typical day of work on a scale from 0 to 10 was 6.12 (S + P = 6, 91; SO = 6.80, *R* = 5.54). Stress translated into psychosomatic symptoms: during the last month, the most frequently reported symptoms included irritability (84 %) and tension (74 %). Emotional exhaustion occurred among 59 %, gloom 44 %, and permanently lowered mood/ depression affected 17 %. Forty-five percent of physicians reported headaches, 25 % sleep disorders, and 9 % eating disorders. Excessive bureaucratization, overwork and haste, with the disparity between effort and compensation are the most common sources of stress. In the ranking of stressors, these preceded the restrictions imposed by the national health service or material shortages and old/ obsolete equipment, or a sense of helplessness in the face of suffering or death. These problems present in the work environment affected doctors to the point of neglecting their personal needs: the most overlooked were rest and sports, together with social and family relationships which were also significantly reduced. Fifty-one percent admitted that the problems experienced at work had a negative impact on their personal lives. This group is characterized by significantly higher levels of stress (average 6.71 to 5.50, Student’s *t* test = 2.712, df 67, *p* < 0.05), neglecting the need to rest (*U* = 408, *p* < 0.05), and the difficulty of finding a proper balance between work and family responsibilities (*U* = 406, *p* < 0.05). Excessive emotional involvement with an oncological patient occurred to varying degrees among 77 to 45 % of the respondents. Eighty-one percent had experienced at least once an episode of unjustified guilt associated with treatment failure or self-imposed excessive requirements. Fifty-four percent admitted to still experiencing such tendencies occasionally. This applies to a greater extent to specialists (*U* = 277.6, *p* < 0.05 emotional engagement; *U* = 251.0, *p* < 0.05 guilt).

### Coping Strategies (Fig. 2C)

The most often reported stress reduction methods were as follows: their relationship with family and/or friends (69 %), reading books/ watching movies (66 %), emotional distance from their problems (63 %), and contact with nature (62 %). Only 6 % of physicians used the help of psychotherapy as a means to combat stress. At the same time, 83 % reported that they had the means to counter their negative experience by talking with family or friends. Those who did not, more often reported a difficulty in finding a proper balance between work and family responsibilities (*U* = 177.5, *p* < 0.05). Gradually and over a period of years, the phenomenon of avoiding the patient increased and levels of empathy slightly decreased (declared by 57 %), and effective communication with the patient subjectively improved. Attitudes such as cynicism, dehumanization of the patient, or a patronizing approach were all rarely reported. Approach to the treatment of the patient as a motivating challenge (48 %) is more than twice as common as treating the patient as a hassle or a cumbersome problem (22 %). Assertiveness and teamwork skills significantly improved; however, unfortunately, 30 % of doctors admit to working at the expense of neglecting their own personal needs. When communicating bad news to the patient, 47 % of doctors revealed in-depth details only at the request of the patient, 35 % tried to communicate sad news sparingly, 18 % had a rule of communicating all information at once with all the details. Seventy-two would respond positively to the proposal of participating in a workshop aimed at improving communication skills and doctor-patient psychological support. The length of the internship had no statistically significant effect on the response given.

### Job Satisfaction (Fig. 2D)

Ninety-six of physicians were satisfied with their decision to pursue work with cancer patients. However, as many as 49 % of oncologists experienced moments of doubt regarding their sense of calling. In their practice, 81 % of physicians noticed the gratitude of patients and their families (most R: 87 %, while 30 % of the S & P did not see it). Work atmosphere is positively assessed by 62 % of doctors, and those who more often described it as unsatisfactory, often pointed to time limitations (*U* = 284, *p* < 0.05) and conflicts within the team (*U* = 244, *p* < 0.05), and felt significantly higher stress levels (average 6.82 to 5.76, *t* test = −2.141, df 66, *p* < 0.05).

## Discussion

The prevalence of symptoms associated with stress disclosed in a study is to be expected. Although the number of oncology professionals with burnout symptoms reported by studies varies (ESMO Congress study of 2014: 71 %, meta - analysis from 2007 covering 10 studies estimates the numbers at 8–51 %) [1, 4] reported problems, symptoms, and reasons remain similar. The problems result from both the discrepancy between the idealized model of work and the reality of work and the above specifics of working with oncological patients. The impact these problems have on stress levels are confirmed by other studies, regardless of the place of the study, with usually lower but still significant numbers of specialists experiencing burnout symptoms. In a Canadian study conducted in Ontario, as many as one third of oncological specialists reported that they considered leaving the job, with an estimated one third experiencing burnout symptoms [5]; a study conducted in the USA in 2003 estimated that the percentage of burned-out oncology physicians exceeded 60 % [6]. The most commonly reported symptoms were, just as in our study, frustration and emotional exhaustion [6]. In a recent (2010) French study, the major components of burnout were reported by 26 % of radiation oncology specialists and 35 % of hematologists [7]. In Australia, high levels of emotional exhaustion were present among one third of oncology health professionals, despite the fact that they possessed high levels of personal accomplishment [8]. Physicians surveyed are also exposed to a number of aggravating factors not only existential in nature but also connected to the organizational constraints of health care. In Australia, the most reported perceived cause of burnout are excessive workload (32.8 %) and frustration with hospital administration and management (22 %), while in the USA overwork and a lack of time were commonly mentioned [6, 8]. Those results do not contradict our study, where excessive bureaucratization, overwork, and haste were also the most often reported contributing factors. Similarly, in the same research, those specialists who did not feel adequately rewarded experienced burnout symptoms more often than others [7]. The problem of “high demand-low influence”—described by M. Dorfmuller explains the frustration arising from the need for following a hospital protocol that collides with the myth of the doctor as a decision maker [9]. Despite the fact that it cannot be entirely ruled out that the problem may be a local issue, the repeatability of results reported by various surveys makes this eventuality highly unlikely.

## Conclusions

Oncologists are not only exposed to a multitude of negative factors which are psychological in nature but they also carry the burden of the organizational limitations of the health service. It cannot be ruled out that in the presented study, this problem may be local in nature. The vast majority of doctors are satisfied with their speciality of choice, despite the difficulties. This may be explained by the original motivation for choosing the particular specialty—often aimed at helping the sick. Young doctors considering pursuing an oncological speciality should not be discouraged by the likely degree of sacrifice or burden, but rather aim to develop effective ways to reduce stress, along with remembering their own health needs. This could be valuable part of both pregradual and postgradual medical education worth to become part of medical *curriculum*.

## Limitations

Because of the small study group and the disproportionate distribution of participants within the studied speciality and seniority groups, a sampling error cannot be ruled out. The limited amount of collected data regarding demographics is another limitation, as the study does not take into account different working conditions, additional activities, or family status. A cross-shot of these problems and the possibility for a variety of interpretations of the questions asked limit the possibility of data analysis.
